# Mirabegron: The most promising adipose tissue beiging agent

**DOI:** 10.14814/phy2.14779

**Published:** 2021-03-02

**Authors:** Jocelyn S. Bel, T.C. Tai, Neelam Khaper, Simon J. Lees

**Affiliations:** ^1^ Biotechnology Program Lakehead University 955 Oliver Road Thunder Bay ON Canada; ^2^ Northern Ontario School of Medicine 955 Oliver Road Thunder Bay, ON and 935 Ramsey Lake Road Sudbury ON Canada; ^3^ Biology Laurentian University 935 Ramsey Lake Road Sudbury ON Canada; ^4^ Chemistry and Biochemistry Laurentian University 935 Ramsey Lake Road Sudbury ON Canada; ^5^ Biomolecular Sciences Program Laurentian University 935 Ramsey Lake Road Sudbury ON Canada; ^6^ Biology Lakehead University 955 Oliver Road Thunder Bay ON Canada

**Keywords:** Brown Adipose Tissue, Browning, Obesity, β3‐Adrenergic Receptor Agonist

## Abstract

Accumulation of white adipose tissue (WAT) underlies the obesity epidemic, leading to current therapeutic techniques that are being investigated for their ability to activate/“beige” this tissue. Adipose tissue (AT) beiging has been reported through intermittent cold exposure (CE), exercise, and β3‐Adrenergic Receptor (β3AR) agonists. But how AT beiging can help in the treatment of metabolic disorders like obesity and type 2 diabetes (T2D) remains largely unexplored. This review summarizes recent research on the use of β3AR agonist, mirabegron (Myrbetriq®), in stimulating beiging in AT. Researchers have only recently been able to determine the optimal therapeutic dose of mirabegron for inducing beiging in subcutaneous/ inguinal WAT, where the benefits of AT activation are evident without the undesired cardiovascular side effects. To determine whether the effects that mirabegron elicits are metabolically beneficial, a comparison of the undisputed findings resulting from intermittent CE‐induced beiging and the disputed findings from exercise‐induced beiging was conducted. Given the recent *in vivo* animal and clinical studies, the understanding of how mirabegron can be metabolically beneficial for both lean and obese individuals is more clearly understood. These studies have demonstrated that circulating adipokines, glucose metabolism, and lipid droplet (LD) size are all positively affected by mirabegron administration. Recent studies have also demonstrated that mirabegron has similar outcomes to intermittent CE and displays more direct evidence for beiging than those produced with exercise. With these current findings, mirabegron is considered the most promising and safest β3AR agonist currently available that has the potential to be used in the therapeutic treatment of metabolic disorders, and future studies into its interaction with different conditions may prove to be useful as part of a treatment plan in combination with a healthy diet and exercise.

## Graphical abstract

Based on the information provided in this review, mirabegron warrants further investigation with respect to adipose tissue beiging and its potential clinical use for the treatment of metabolic disorders in combination with a healthy diet and exercise.

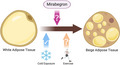

## 1 INTRODUCTION

Adipose tissue (AT) is an emerging tissue of interest in the area of metabolic‐related research due to its ability to act as a storage organ (white adipose tissue (WAT)) and a regulator of thermogenesis (brown adipose tissue (BAT)). Metabolically active BAT has been positively correlated to improved energy, glucose, and whole body metabolism (Herz & Kiefer, 2019). BAT can be activated from a wide range of stimuli including cold temperatures, pharmacological compounds, exercise, and even amino acids (Herz & Kiefer, 2019; Huska et al., 2020). We have recently demonstrated that essential amino acid leucine can potentiate the effects of glucose on BAT, as shown by increased ^18^F‐FDG uptake, and further promote positive health effects (Huska et al., 2020). With the growing interest in this tissue, the ability to stimulate WAT to become “beige” has been investigated by researchers as activated beige AT will act less like WAT and function more like BAT, which produces heat, can contribute to thermogenesis and act as an endocrine organ improving glucose metabolism. AT is known to play key roles in regulating metabolism and the appearance of beige AT in conventional WAT depots could aid in combating obesity and other metabolic disorders. The method conventionally used to study this mechanism involves exposing subjects to cold temperatures for varying amounts of time. However, this method also has limitations as the stimulation of the sympathetic nervous system (SNS) releasing catecholamines (norepinephrine (NE) and epinephrine from sympathetic nerve terminals and the adrenal medulla, respectively), thus rendering its effects in the body and is systematically difficult to control. Moreover, mandating participants to enter cold exposures (CE) for long or intermittent periods is difficult to follow and will likely have limited compliance from the participants. This exact reason has led to the recent investigation into the utilization of pharmacological compounds that can mimic the sought aftereffects of CE on AT.

Beiging, either through CE or β3AR agonists, has been linked to metabolic improvements, namely through increased glucose tolerance and insulin sensitivity (Baskin et al., 2018; Carpentier et al., 2018; Chen et al., 2020; Cypess et al., 2015; Darcy & Tseng, 2019; Finlin et al., 2018; Hao et al., 2019; Herz & Kiefer, 2019; Jiang et al., 2017; Loh et al., 2019; Marlatt & Ravussin, 2017; O’Mara et al., 2020; Pan et al., 2020; Peres Valgas da Silva et al., 2019; Phillips, 2019; Singh et al., 2020; Srivastava & Veech, 2019; Wang et al., 2019). Activation of the β3AR by NE on BAT initiates a cascade of secondary messengers within the cell. These messengers will lead to the stimulation of the uncoupling protein 1 (UCP1), a protein classically associated with BAT, that will uncouple the energy production from the electron transport chain and use stored fatty acids to produce heat. However, UCP1 has also been found in stimulated beige AT which has propelled the idea of beiging to the forefront of AT science.

The use of pharmacological compounds in lieu of prolonged periods of CE to stimulate the beiging of AT is gaining more interest in an attempt to mitigate and treat metabolic disorders. A combination of exercise and a healthy diet, with a β3AR agonist, may be a promising addition to metabolic disorder treatment plans. Many compounds ranging from naturally occurring antioxidants to hormones, to various β3AR agonists have been investigated. The most promising results are found with β3AR agonists, although many of them are accompanied by undesirable cardiovascular side effects (Baskin et al., 2018; Carpentier et al., 2018; Chen et al., 2020; Cypess et al., 2015; Finlin et al., 2018; Hao et al., 2019; Herz & Kiefer, 2019; Loh et al., 2019; Marlatt & Ravussin, 2017; O’Mara et al., 2020; Pan et al., 2020; Pouleur et al., 2018; Singh et al., 2020). β3AR agonists and their possible use in combating metabolic disorders has previously been discussed elsewhere (Chen et al., 2020; Cypess et al., 2015; Marlatt & Ravussin, 2017; Michel et al., 2010; Schena & Caplan, 2019). The one pharmacological compound that shows both the promising effects of beiging and minimal cardiovascular side effects is mirabegron. Mirabegron (Myrbetriq®) was approved by the U.S. Food and Drug Administration in 2012 for the treatment of overactive bladder at doses of either 25 or 50 mg/day. The bladder is also lined with β3ARs, which are targeted by mirabegron to allow for the smooth muscles to relax and therefore increase the storage of urine (Myrbetriq, 2012). With β3ARs also found in BAT and inducible beige AT, this agonist illustrates the ability to activate AT and allow for positive metabolic outcomes (discussed below) without negative cardiovascular side effects. With mirabegron being the most promising pharmacological compound available to increase AT beiging, this review will focus on the most recent clinical and pre‐clinical research advances found within the field.

### 1.1 Impacts of mirabegron on adipose tissue

The impact of mirabegron on AT and beiging will be affected by noted differences between rodents and humans with respect to AT depots and the amount of BAT. Rodent BAT remains throughout its life and has been conventionally studied from the interscapular or subscapular regions (Mo et al., 2017). Mice also have smaller BAT regions being around the kidneys (perirenal BAT), aorta (mediastinal BAT), or between the neck and head (cervical BAT) (Mo et al., 2017). While human BAT was previously thought to disappear after infancy, it is now known to persist throughout life and is mainly located in the supraclavicular (scBAT) region (Mo et al., 2017). While other human BAT locations can also be found surrounding the kidneys and the heart, the direct depot‐to‐depot comparison of rodent interscapular BAT (iBAT) disappears in humans as they age (Liu et al., 2017; Mo et al., 2017). This lack of depot‐to‐depot equivalency in adult humans and mice presented the most common concern with rodent BAT research and its translation to humans (Mo et al., 2017). Mo et al. (2017) were the first to attempt to close this gap and illustrated that the scBAT depot found in mice has similar thermogenic potential as iBAT but is also analogous to the scBAT region in humans. Through morphological characteristics, protein and gene expressions, and location, this scBAT depot will be useful for BAT studies that are translatable from mice to humans (Mo et al., 2017). At the receptor level, the mouse β3AR is 81% identical to that in humans, with the greatest homology (94%) occurring in the transmembrane domains of the receptor (Schena & Caplan, 2019). A review by Warner and Mittag (2016) provided comparisons between human and rodent AT in the context of beiging. One possible explanation for the differences observed in the beiging responses of rodents and humans could be related to the sensitivity of the β3AR itself, with human β3AR thought to be less sensitive than those in rodents (Warner & Mittag, 2016). Even with differences in the β3ARs, it is accepted across the scientific community that agonists for these receptors are metabolically beneficial in humans, but more research into how these agonists, such as mirabegron, truly add value to whole body metabolic processes in humans (Michel et al., 2010; Schena & Caplan, 2019; Warner & Mittag, 2016).

Mirabegron has shown tremendous promise in stimulating AT to improve metabolism and mitigate metabolic disorders. Mirabegron administration to both lean and obese humans and rodents fed a high‐fat diet (HFD) have been investigated and compared with CE treatments in order to test the efficacy of mirabegron. To date, no studies have reported the same level of negative side effects found with other β3AR agonists, and mirabegron's effectiveness in positively improving metabolism continues to be explored by researchers.

Loh et al. (2019) reported a dose‐response study with mirabegron and its effect on skin temperature, blood pressure (BP), and heart rate (HR) in healthy male and female participants. They investigated the effects of mirabegron on these measurements at four different doses: 50, 100, 150, and 200 mg/day. Mirabegron was delivered as a single oral dose for 4 days with a wash‐out period ranging from 3 to 14 days between doses (Loh et al., 2019). The effects were dose‐dependent, but a dose of 100 mg/day increased the skin temperature and energy expenditure (EE) without any cardiovascular side effects such as increased BP and HR that was found with the 150 and 200 mg doses, respectively (Loh et al., 2019). This study gave insight into the dosage of mirabegron that healthy participants can withstand where increase in EE and skin temperature (indicative of BAT activation) can be observed. This was an important study to show that a higher dose of mirabegron than administered for overactive bladder could be tolerated and further illustrated that beiging can be observed without negative side effects. All other studies previously conducted on beiging with high doses of mirabegron (>150 mg/day) were accompanied by negative cardiovascular side effects. O’Mara et al. (2020) further verified these findings as they also found that a mirabegron dose of 100 mg/day can increase the metabolic activity of BAT and improve metabolic parameters, such as glucose uptake and insulin sensitivity. This study investigated the effects of 100 mg/day of mirabegron over four weeks in healthy women (O’Mara et al., 2020). The metabolic activity of BAT and resting EE increased in the participants; however, the overall bodyweight was not affected (O’Mara et al., 2020). Glucose metabolism was also significantly improved with participants showing both higher insulin sensitivity (36% increase) and secretion that was accompanied by a 34% increase in glucose effectiveness (O’Mara et al., 2020). The circulating levels of adiponectin, high density lipoprotein, and apolipoprotein A‐1 were also increased after the four‐week treatment, further illustrating the beneficial metabolic properties of mirabegron (O’Mara et al., 2020). Even with brown and beige adipocytes not uniquely identified in this study, it is likely that both adipocyte types contributed to a higher metabolic activity with mirabegron administration (O’Mara et al., 2020). Finlin et al. (2020) recently conducted a clinical trial with mirabegron administration (50 mg/day for 12 weeks) in obese, insulin‐resistant men (Finlin et al., 2020). With mirabegron administration, beiging in the inguinal WAT (ingWAT) was increased, and the subjects had significantly improved glucose homeostasis and insulin resistance (Finlin et al., 2020). Moreover, this was accompanied by reduced AT dysfunction, as indicated with the presence of alternatively activated macrophages (Finlin et al., 2020). This new study further proves how mirabegron shows promise as a beiging agent in humans (Finlin et al., 2020).

The effects of mirabegron on obesity were studied by Hao et al. (2019) where male C57BL/6 J mice were fed a HFD for 7 weeks prior to mirabegron (2 mg/kg/day) being delivered through osmotic pumps. Mirabegron increased UCP1 protein expression, lowered bodyweight gain, decreased adiposity, and altered the lipid droplet (LD) size in BAT to be much smaller in these obese mice (Hao et al., 2019). The beiging capabilities of mirabegron were also noted with the appearance of beige adipocytes in ingWAT, even in mice fed an HFD. Glucose metabolism was also altered with increased glucose tolerance and improved insulin sensitivity with mirabegron administration (Hao et al., 2019). The findings in this study illustrate the mitigating effects of mirabegron on high‐fat diet‐induced obesity.

Chen et al. (2020) reviewed the idea of thermogenesis and increased EE for managing obesity and noted that the currently approved dose of mirabegron (50 mg/day) is not enough to increase the EE in humans (Chen et al., 2020). Baskin et al. (2018) found that mirabegron was able to induce WAT lipolysis, and BAT thermogenesis in humans; however, they only tested two doses of 50 and 200 mg/day, where only the 200 mg dose increased BAT activation and resting EE (Baskin et al., 2018). The negative cardiovascular side effects and off‐target binding to the β1‐Adrenergic Receptor (β1AR) were also found with the 200 mg/day dose, and not observed at the 50 mg dose (Baskin et al., 2018). This illustrates how the metabolic benefits of mirabegron needs to be balanced with the negative cardiovascular side effects that are observed with higher doses.

Recent advances in understanding how to combat obesity with thermogenesis was reviewed by Pan et al. (2020). They described how mirabegron can increase BAT uptake of ^18^F‐FDG and non‐esterified fatty acids, indicating its promising effects in the pursuit of beiging (Pan et al., 2020). However, no β3AR agonist has been approved for stimulating beiging yet and the possibility of increased BP and plaque development due to the off‐target binding of mirabegron in the myocardium and blood vessels are still being investigated (Pan et al., 2020). These negative effects appear to be dose‐dependent or appear when a patient has an underlying heart condition (discussed below).

### 1.2 Cold exposure vs. mirabegron

With CE being the natural stimulant for BAT and beige AT, it is no surprise that lower temperatures show profound effects on these fat depots. The unknowns lie with how mirabegron compares to CE for beige AT activation. Research into comparing the effects of mirabegron and CE on human beige AT has been conducted in both male and female participants who were either classified as lean or obese (Finlin et al., 2018). The participants were either exposed to a 30‐minute ice pack application on the upper thigh for 10 days or treated with mirabegron (50 mg/day for 10 weeks) (Finlin et al., 2018). After CE, the subcutaneous WAT had significant increases in both UCP1 and transmembrane protein 26 (over a threefold change) in both patient body types (Finlin et al., 2018). Mirabegron also increased these two proteins in obese subjects (Finlin et al., 2018). The appearance of beige AT in lean participants is inducible in subcutaneous WAT by both CE and mirabegron, and obese participants are also able to have increases in beige AT (Finlin et al., 2018). While mirabegron treatment did not show increases in PGC‐1α, an important protein related to beiging, this may have been the result of the increased immune cells present in obese AT, thereby inhibiting PGC‐1α (Finlin et al., 2018). Without PGC‐1α, the beiging process may be somewhat limited in obese subjects, but the ability to beige obese, insulin‐resistant subjects found in this study cannot be undervalued (Finlin et al., 2018). PGC‐1α may be targeted with other medications or diet in combination with mirabegron in future studies, which can lead to even more beiging within these obese, insulin‐resistant subjects (Finlin et al., 2018).

Healthy individuals who take mirabegron have increased WAT lipolysis and BAT activation that is similar to matched CE counterparts (Cypess et al., 2015). Marlatt and Ravussin (2017) reviewed the topic of BAT activation leading to a healthier life and found that increased resting metabolic rates (RMR) was observed when BAT was stimulated with either CE or mirabegron by over 100 and 200 kcal/day increases, respectively (Carpentier et al., 2018; Cypess et al., 2015; Marlatt & Ravussin, 2017). The improved metabolic rate accompanied by increased glucose uptake (as measured by ^18^FDG) in BAT indicates that mirabegron can elicit similar effects as CE (Darcy & Tseng, 2019). The possibilities with mirabegron activated beige AT were compared with other β3AR agonists by Singh et al. (2020) with transplanted human beige adipocytes in a mouse model. Using high throughput screening, they tested a range of compounds and found that mirabegron was the only agonist able to activate both the thermogenic program and lipolysis in these human beige adipocytes (Singh et al., 2020). With mirabegron illustrating the most positive results for β3AR activation and associated metabolic outcomes, the agonist's ability to influence the inflammatory response associated with metabolic disorders was also investigated by the researchers (Jarc & Petan, 2020; Kälin et al., 2017). The association between AT T‐cell tolerance and CE were studied using humanized mice treated with mirabegron for 3 days (1 mg/kg/day) (Kälin et al., 2017) and their findings of improved regulatory T‐cell induction further illustrates promising trends for combating metabolic disorder associated tissue inflammation (Jarc & Petan, 2020; Kälin et al., 2017). The beiging effects and improved metabolic profiles found with mirabegron cannot be disputed; however, the accompanying negative cardiovascular side effects observed at high doses are a precaution that researchers have been investigating in the last few years.

### 1.3 Cardiovascular safety of mirabegron

One possible side effect of Myrbetriq® is increased BP, especially if a patient has a history of high BP (2012). These negative cardiovascular side effects, found most predominantly in the 200 mg/day dose, have been hypothesized to arise from off‐target binding to the β1AR in the heart (Baskin et al., 2018). However, this off‐target binding may be mitigated if β1AR agonists are administered in conjunction with mirabegron (Cypess et al., 2015).

Not all associations between mirabegron and the heart have been negative. Since mirabegron's approval, clinical trials conducted by Chapple et al. (2013) and Nitti et al. (2014) on its effectiveness in patients with overactive bladder have both concluded that there is a low association between mirabegron and adverse cardiovascular events (Chapple et al., 2013; Nitti et al., 2014). Jebessa et al. (2020) studied β3ARs in the heart and noted that some agonists may possess cardioprotective role in regard to lipid accumulation; however, further clinical studies with mirabegron in participants with structural heart disease will give insight into how this agonist functions. There is currently a clinical trial being conducted examining the direct effects of mirabegron on participants with heart disease to tackle this gap in the literature (Jebessa et al., 2019; Pouleur et al., 2018). Further investigations into the interactions between β3ARs located other than the AT are critical for future therapeutic uses (Wang et al., 2019).

### 1.4 The beiging effects of exercise vs. mirabegron

Exercise has always been a part of a healthy lifestyle, and physical activity is vitally important in patients with metabolic disorders. In addition to a healthy diet, exercise allows for improved glucose tolerance and insulin sensitivity (Peres Valgas da Silva et al., 2019). During exercise, WAT will supply stored triglycerides that will allow for the body to expend more energy (Vidal & Stanford, 2020). The release of triglycerides from AT will lead to metabolic changes including increased mitochondrial activity and thermogenesis, increased glucose uptake, increased lipolysis and lipid metabolism, reduced inflammatory factors, and smaller adipocyte size (Vidal & Stanford, 2020). Exercise itself has been investigated for its effects on AT beiging and has been reviewed previously (Aldiss et al., 2018; Dewal & Stanford, 2019; Peres Valgas da Silva et al., 2019; Phillips, 2019; Srivastava & Veech, 2019; Vidal & Stanford, 2020).

When comparing the effects of CE and exercise on beiging of ingWAT in rodents, CE lowers tissue mass, whereas exercise fails to induce this change (Chung et al., 2017). CE ultimately leads to increased glucose uptake in BAT, while exercise promotes glucose uptake primarily in the skeletal muscles. Increased glucose utilization in skeletal muscles illustrates the most profound effect on metabolic health, regardless of BAT activation, which proves that the benefits of exercise are independent of BAT activity (Geng et al., 2019; Peres Valgas da Silva et al., 2019; Srivastava & Veech, 2019). Our group has recently reported that hepatic insulin resistance can be prevented with voluntary physical activity, demonstrating a link between increased energy utilization in skeletal muscles during physical activity to improve whole body glucose metabolism (Sarvas et al., 2015). The connection between exercise and improved glucose metabolism are generally accepted; it is unclear however how AT thermogenic activity and beiging are directly implicated in this process.

There are still unknowns when it comes to understanding how exercise affects AT thermogenic activity as some studies have reported decreased BAT activity and glucose uptake in response to exercise, while others have reported the opposite (Peres Valgas da Silva et al., 2019; Pouleur et al., 2018; Vidal & Stanford, 2020). Exercise induces a whole body response with many effects including transient increases in fatty acids and catecholamines, which could also increase AT beiging, further adding to the complexity of exercise‐induced beiging. WAT studies investigating the effects of exercise have shown reductions in adipocyte size, increased mitochondrial activity, and alterations in gene expressions, but the mechanisms for these changes are not well understood (Dewal & Stanford, 2019; Vidal & Stanford, 2020). Vidal and Stanford (2020) recently reviewed exercise‐induced adaptations in AT and highlighted the differences found between humans and rodents when it comes to exercise‐induced beiging. They also suggest that beiging may not be a direct result of exercise, but rather an indirect result of other stimuli (Vidal & Stanford, 2020).

In beige AT, there are conflicting results when comparing the effects of exercise, especially in lean and obese rodents. In lean rodents, the activation of beige adipocytes through exercise is associated with increased beige genetic markers *Ucp1*, *Prdm16*, and *Pgc1α* (Dewal & Stanford, 2019; Peres Valgas da Silva et al., 2019). However, in HFD‐induced obese rodents, exercise has been reported to either increase, decrease, or not affect these beige markers at all (Peres Valgas da Silva et al., 2019; Srivastava & Veech, 2019). This lack of consensus illustrates a major downfall with exercise‐induced beiging. Two studies conducted by Stanford et al. (2015) and Bostrom et al. (2012) are highlighted to be the ones with the most promising research concerning exercise‐induced beiging (Boström et al., 2012; Dewal & Stanford, 2019; Phillips, 2019; Srivastava & Veech, 2019; Stanford et al., 2015; White et al., 2019). Stanford et al. (2015) illustrated that exercise‐trained rodents had beige adipocytes accompanied by increased mitochondrial activity, beige genes (including *Ucp1*, *Prdm16*, *Pgc1α*, *Tbx1*, *Tmem26*, and *Cd137*), improved glucose metabolism and tissue alterations, all of which improved overall health (Stanford et al., 2015). To further understand these effects, the beige AT from exercised mice were transplanted into matched sedentary mice, who then also gained improved metabolic profiles (decreased circulating glucose and insulin levels) (Stanford et al., 2015). The promising effects of exercise‐induced beige AT activation have been hypothesized to rise from other exercise associated factors such as IL‐6 (Aldiss et al., 2018; Srivastava & Veech, 2019; Stanford et al., 2015; White et al., 2019), irisin (Aldiss et al., 2018; Chung et al., 2017; Sarvas et al., 2015; Srivastava & Veech, 2019; White et al., 2019), Fibroblast Growth Factor 21 (FGF21) (Srivastava & Veech, 2019; White et al., 2019), and Vascular Endothelial Growth Factors (VEGF) (Aldiss et al., 2018; Boström et al., 2012), but further research into these relationships is required. Our laboratory has demonstrated the importance of IL‐6 in mediating improved whole body glucose metabolism in both the gastrocnemius and plantaris muscles (Sarvas et al., 2014). In response to voluntary physical activity, endogenous IL‐6 plays an important role in preventing insulin resistance under a HFD, and this beneficial metabolic effect is absent in IL‐6 KO mice (Sarvas et al., 2014). With this finding in skeletal muscles, the same outcome may also be found in AT and may be related to the beiging process. Bostrom et al. (2012) illustrated the effects of irisin on UCP1 expression in beige AT in response to exercise in mice and found that exercise‐induced increases in this circulating factor can reduce diet‐induced obesity and insulin resistance by inducing beiging (Boström et al., 2012). Geng et al. (2019) illustrated how exercise can improve metabolic dysfunction by enhancing the effects of FGF21 within the AT of HFD induced male mice (Geng et al., 2019). With exercise training 5 days/week for 4 weeks, there was a decrease in body weight, increased sensitivity of AT to FGF21, and improved metabolic homeostasis (Geng et al., 2019; Peres Valgas da Silva et al., 2019). These studies provide insight into how the systematic effects of exercise can relate to beiging and therefore lead to better AT health, but further research is needed to understand whether these findings can be replicated in humans and how exactly this pathway works.

Some concerns have been raised when it comes to exercise being used to induce beiging since the frequency, intensity, and duration of exercise, in addition to the participant's age, sex, and lifestyle are factors that vary between studies, making conclusions difficult to compare (Jebessa et al., 2019; Pouleur et al., 2018). With these variables also present in human populations, diet and exercise may not be enough to promote a healthy lifestyle in every patient with a metabolic disorder. This is especially true with insulin‐resistant, obese subjects (Darcy & Tseng, 2019; Finlin et al., 2018; Jiang et al., 2017). Compared with other avenues for inducing beiging in WAT, such as with mirabegron or CE, exercise does not show the same effect on the β3ARs, leaving it less likely to be a beiging agent (Darcy & Tseng, 2019; Finlin et al., 2018; Jiang et al., 2017). Despite this limitation, it is reasonable to suggest that a combination of diet, exercise and possibly a β3AR agonist, may provide benefits for better metabolic health and should be further investigated in future clinical trials. While no drug will replace exercise, mirabegron in concert with increased physical activity may provide complementary effects on whole body metabolism.

## 2 SUMMARY & CONCLUSION

Many studies have reported that mirabegron or other selective β3AR agonists could be useful in treating metabolic disorder in humans, but more insight into these compounds and their side effects is required. This review highlights the most recent findings in the understanding of mirabegron for the use of AT activation and overall health. Understanding the impact of varying doses and genetic properties that contribute to pathological cardiovascular effects previously observed with mirabegron studies, future research can be better situated to overcome these issues. Since AT contributes to whole body metabolism, its chronic activation could help mitigate the pathological effects of metabolic disorder. Many of the metabolic effects of having increased BAT activity, including protection from insulin resistance, reduced adiposity, a “metabolic sink” for glucose and fatty acids, in addition to thermogenesis all contribute to the whole body health and decrease the potential of age‐related diseases including cancer, diabetes, cardiovascular disease, and dementia (Darcy & Tseng, 2019). With improved plasma parameters, BAT activation, and beiging capabilities in WAT, this pharmacological compound is a promising option for stimulating the β3ARs within AT. In combination with diet and exercise, mirabegron could prove to be a helpful tool in slowing down the expanding obesity epidemic (Wang et al., 2019). When administered to healthy individuals, mirabegron does not seem to significantly alter body weight but can still improve metabolism and activate AT. In obese individuals, or rodents fed HFDs, mirabegron appears to have a greater effect on AT lipolysis and LD size contributing to its activation and whole body metabolism (Baskin et al., 2018; Chen et al., 2020; Finlin et al., 2018; Hao et al., 2019; Loh et al., 2019; O’Mara et al., 2020). Due to the potential benefits it could provide and the low risk of adverse cardiac events, it is feasible to conduct further clinical trials with mirabegron in the stimulation of beige AT. Administration of mirabegron and the stimulation of beige AT will be advantageous in treating insulin resistance, glucose intolerance, and metabolic disorders. Based on the information provided in this review, mirabegron warrants further investigation with respect to AT beiging and will be a useful addition in the treatment plan for metabolic disorders in combination with a healthy diet and exercise.

## AUTHOR CONTRIBUTION

JSB, SJL, NK, and TCT conceived the ideas in the manuscript. JSB wrote the short review. SJL, NK, and TCT critically revised the article for important intellectual content and flow. All authors approved the final version of the manuscript and agree to be accountable for all aspects of the work in ensuring that questions related to the accuracy or integrity of any part of the work are appropriately investigated and resolved.
